# The Randomised Evaluation of early topical Lidocaine patches In Elderly patients admitted to hospital with rib Fractures (RELIEF): feasibility trial protocol

**DOI:** 10.3310/nihropenres.13438.1

**Published:** 2023-07-27

**Authors:** Amanda Lewis, Madeleine Clout, Jonathan Benger, Philip Braude, Nicholas Turner, James Gagg, Emma Gendall, Simon Holloway, Jenny Ingram, Rebecca Kandiyali, Nick Maskell, David Shipway, Jason E Smith, Jodi Taylor, Alia Darweish-Medniuk, Edward Carlton

**Affiliations:** 1 Bristol Trials Centre (BTC), Population Health Sciences, Bristol Medical School, University of Bristol, Bristol, England, UK; 2 Emergency Care, Faculty of Health and Applied Sciences, University of the West of England, Bristol, England, UK; 3 Department of Elderly Care Medicine, Southmead Hospital, North Bristol NHS Trust, Bristol, England, UK; 4 Department of Emergency Medicine, Musgrove Park Hospital, Taunton, England, UK; 5 Research and Innovation, Southmead Hospital, North Bristol NHS Trust, Bristol, England, UK; 6 Pharmacy Clinical Trials and Research, Southmead Hospital, North Bristol NHS Trust, Bristol, England, UK; 7 Population Health Sciences, Bristol Medical School, University of Bristol, Bristol, England, UK; 8 Warwick Clinical Trials Unit, Warwick Medical School, University of Warwick, Warwick, England, UK; 9 Academic Respiratory Unit, Translational Health Sciences, Bristol Medical School, University of Bristol, Bristol, England, UK; 10 Emergency Department, Derriford Hospital, University Hospitals Plymouth NHS Trust, Plymouth, UK; 11 Department of Anaesthesia and Pain Medicine, Southmead Hospital, North Bristol NHS Trust, Bristol, England, UK; 12 Department of Emergency Medicine, Southmead Hospital, North Bristol NHS Trust, Bristol, England, UK; 13 Translational Health Sciences, Bristol Medical School, University of Bristol, Bristol, England, UK

**Keywords:** Randomised Controlled Trial, Feasibility, Lidocaine patch, Rib fractures, Frailty

## Abstract

**Background:**

Topical lidocaine patches, applied over rib fractures, have been suggested as a non-invasive method of local anaesthetic delivery to improve respiratory function, reduce opioid consumption and consequently reduce pulmonary complications. Older patients may gain most benefit from improved analgesic regimens yet lidocaine patches are untested as an early intervention in the Emergency Department (ED). The aim of this trial is to investigate uncertainties around trial design and conduct, to establish whether a definitive randomised trial of topical lidocaine patches in older patients with rib fractures is feasible.

**Methods:**

RELIEF is an open label, multicentre, parallel group, individually randomised, feasibility randomised controlled trial with economic scoping and nested qualitative study. Patients aged ≥ 65 years presenting to the ED with traumatic rib fracture(s) requiring admission will be randomised 1:1 to lidocaine patches (intervention), in addition to standard clinical management, or standard clinical management alone. Lidocaine patches will be applied immediately after diagnosis in ED and continued daily for 72 hours or until discharge. Feasibility outcomes will focus on recruitment, adherence and follow-up data with a total sample size of 100. Clinical outcomes, such as 30-day pulmonary complications, and resource use will be collected to understand feasibility of data collection. Qualitative interviews will explore details of the trial design, trial acceptability and recruitment processes. An evaluation of the feasibility of measuring health economics outcomes data will be completed.

**Discussion:**

Interventions to improve outcomes in elderly patients with rib fractures are urgently required. This feasibility trial will test a novel early intervention which has the potential of fulfilling this unmet need. The Randomised Evaluation of early topical Lidocaine patches In Elderly patients admitted to hospital with rib Fractures (RELIEF) feasibility trial will determine whether a definitive trial is feasible.

**ISRCTN Registration:**

ISRCTN14813929 (22/04/2021).

## Plain English summary

Background and aims: Patches containing a numbing medication (lidocaine), put on the skin over broken ribs, may help to improve outcomes in older people. We will carry out a clinical trial of these patches, to see whether this research would work in Accident and Emergency (A&E) and whether patients will take part.

Design: We will ask 100 older people who need to stay in hospital with broken ribs to take part in this research. We will ask permission from relatives to include people with dementia. We will put those who agree to take part into one of two groups by chance. One group will have the patch put over their broken ribs in A&E for up to 3 days, along with usual pain killers if needed. We will treat people in the other group in the normal way, without a patch. We will track how many people are willing to take part. We will collect information on patient recovery in the 30 days after going to A&E and ask people to complete questionnaires about their health. We will interview patients and clinicians to get feedback.

Patient and Public Involvement: Patient volunteers helped us design this research and will provide advice throughout. They agreed that including older people was appropriate, people with dementia and their carers should take part, and side-effects of strong pain killers are important to patients.

Findings: We will use the research findings to develop a larger trial to see if lidocaine patches help patients with broken ribs. We will write up results for scientific journals, speak at conferences and to our patient group.

## Introduction

Trauma in older patients is increasingly recognised as a significant challenge to healthcare systems ^1^. In England, over 20,000 older patients are admitted to hospital each year having sustained injury ^2^. Rib fractures represent the most common non-spinal fracture in older people ^3^. Advancing age is a predictor of morbidity and mortality in patients with rib fractures ^4^. Pain from rib fractures can compromise normal respiratory function, resulting in lung collapse and respiratory compromise. As a result, over 15% of older patients with rib fractures suffer from pulmonary complications, including pneumonia and death ^4^. Older patients who suffer from pulmonary complications have an increased hospital length of stay, with one study demonstrating a mean increase in length of stay of nine days ^5^.

The mainstay for treatment of rib fracture pain is strong opioid analgesia, such as morphine. However, as a result of poor physiological reserve, older patients are vulnerable to the side effects of strong opioid medication such as nausea, constipation, sedation and delirium and respiratory depression ^6^. Invasive approaches, such as thoracic epidural anaesthesia and peripheral nerve blockade, have been used to reduce the likelihood of these side effects. Such approaches require specialist anaesthetic support, monitoring in a high-dependency environment and are only utilised in around 20% of patients admitted with rib fractures ^7, 8^

Topical lidocaine patches, applied over rib fractures, have been suggested as a non-invasive method of local anaesthetic delivery to improve respiratory function, reduce opioid consumption and consequently reduce pulmonary complications ^9^. Lidocaine patches have been shown to be effective and safe in the treatment of other pain, such as post-herpetic neuralgia (shingles) ^10^. Numerous studies have evaluated safety of the patches and no clinically significant systemic adverse effects have been noted, including when used in an elderly population ^11^. Studies have evaluated the use of lidocaine patches in patients with rib fractures, showing reductions in opioid use ^12^, improvements in pain scores ^13, 14^ and length of stay ^15^. However, these studies are generally limited by retrospective design and low patient numbers with consequent bias and low precision. Importantly, none has focussed on older patients who stand to gain most benefit from improved analgesic regimens or tested lidocaine patches as an early intervention in the Emergency Department (ED), when opioid analgesia is still the mainstay of treatment.

There remain a number of uncertainties around conducting a trial evaluating lidocaine patches in older patients with rib fractures, in an emergency setting, which necessitate a feasibility trial. There are significant barriers to recruitment of older adults in research relating to substantial health problems, social and cultural barriers, and potentially impaired capacity to provide informed consent ^16^. In addition, recruitment of patients with frailty, who are in pain, in an emergency setting, may pose challenges around information provision and data collection, including clinical outcomes, pain assessment and patient reported outcomes.

The aim of this trial is to investigate the uncertainties around trial design and conduct to establish whether a definitive trial is feasible and to optimise the design of such a trial. The objective is to test processes and gather information for the planning of a definitive randomised controlled trial (RCT) to evaluate early topical lidocaine patches in older patients admitted to hospital with rib fractures.

This protocol is reported in line with Standard Protocol Items: Recommendations for Interventional Trials (SPIRIT) guidelines.

### Design and conduct

RELIEF is a multicentre, parallel group, individually randomised, feasibility RCT with economic scoping and nested qualitative study. The trial will take place across at least six sites in the UK to ensure demographic spread. We will select Major Trauma Centres that are more likely to see the more severe end of the injury spectrum, and Major Trauma Units that may see patients with less severe injuries.

As confirmed by the Medicines and Healthcare products Regulatory Agency (MHRA), this feasibility trial is not a Clinical Trial of an Investigational Medicinal Product (CTIMP) as defined by the EU Directive 2001/20/EC.

This is an open trial. Participants’ trial allocations will only be blinded to those performing central review of data for the assessment of outcomes where feasible. 

Patient and public involvement was ensured at all stages of trial design, and will continue through a patient advisory group and patient representation on the trial management group (TMG) and trial steering committee (TSC). Day-to-day trial management is administered through the UKCRC-registered Bristol Trials Centre at the University of Bristol and sponsored by North Bristol NHS Trust.

### Trial population

The screening and recruitment of patients, delivery of intervention and recording of outcomes will be carried out within participating EDs in the UK. Full inclusion and exclusion criteria are detailed in Box 1.

**Box 1. RELIEF feasibility trial inclusion and exclusion criteria**

**Inclusion criteria**

1. Older adult patients (age ≥ 65 years).
2. Presenting to the Emergency Department (ED) with traumatic rib fracture(s) (including multiple fractures, flail chest and traumatic haemo/pneumothorax even if this requires intercostal chest drainage and bilateral rib fractures), confirmed radiologically (by chest X-Ray or CT scan conducted as part of routine care).
3. Requiring hospital admission for ongoing care.

**Exclusion criteria**

1. Serious distracting trauma to other body regions (adjudicated by the treating clinician): examples include but may not be limited to: traumatic brain injury with cognitive impairment, acute spinal column fracture or spinal cord injury, abdominal and lower limb injuries requiring surgery, unstable pelvic fracture.
2. Requirement for intubation and mechanical ventilation either prehospitally or in the ED.
3. History of allergy to lidocaine.
4. Open wounds at the site of patch application.
5. End-stage dementia (adjudicated by the treating clinician, e.g. bed-bound and non-verbal).
6. End-stage liver failure with jaundice.
7. End-stage heart failure with breathlessness at rest prior to injury.
8. Those unable to communicate in English language where all reasonable attempts to source translation services are exhausted within the ED.
9. Patients transferred from non-recruiting units to a recruiting site who have a lidocaine patch applied as part of standard care prior to arrival in the recruiting site.

### Screening, recruitment and consent

Hospital staff will complete a trial-specific screening log, which will be developed in line with the SEAR (Screened, Eligible, Approached, Randomised) framework ^17^; this framework will enable us to record the flow of potential participants through the recruitment process, in line with recommended Consolidated Standards of Reporting Trials (CONSORT) reporting guidelines ^18, 19^. Where possible, screening logs will include reason(s) for non-participation.

Recruitment will be undertaken 24 hours a day, 7 days a week. Recruitment by appropriately trained ED clinicians out of normal research nursing hours will be important to assess as part of this feasibility trial. Potentially eligible participants will be identified at the time of arrival in ED by clinical staff or research nurses.

***Consent in patients with capacity.*** During initial assessment, a member of the ED clinical team or research nurse will give the patient the written trial Summary Participant Information Sheet (Summary PIS) and, where appropriate, the full Participant Information Pack. Patients who are willing and eligible to participate in the trial will be asked to provide written informed consent. Written informed consent will not be sought until the patient is comfortable and immediate care needs have been addressed. However, given the intervention is early (applied within the ED), consent will be obtained prior to transfer to the inpatient ward. Ideally patients will have read both the Summary PIS and Participant Information Pack before providing written informed consent. It may not be appropriate or feasible for some patients (e.g. due to being in pain) to read both documents, or at least not the Information Pack in full, prior to providing written informed consent. In these circumstances, patients who have read the Summary PIS and are willing and eligible to participate will be asked to provide full written informed consent and asked to read the Information Pack in full as soon as appropriate.

***Patients who may potentially lack capacity to provide consent for themselves.*** Patients who lack capacity to consent for themselves may be eligible for inclusion. A patient’s cognitive impairment may relate to a neurodegenerative disease, such as dementia, which is likely to remain throughout the duration of the trial due to its chronicity. One third of patients aged over 65-years admitted to hospital after a fall have dementia ^20^. Patients with dementia are more susceptible to both the effects of pain after injury and to the side effects of strong opioid medication traditionally used to treat rib fractures ^21^. There is an increasing recognition that older patients will under-report pain, however, studies evaluating this important issue have failed to address cognitive impairment as a confounder in pain reporting ^8^. Alternatively, a patient’s cognitive impairment may, at initial approach, be due to an acute medical condition or emergency, such as delirium, and may therefore be temporary. Additionally, some participants who have capacity at the time of consent in the ED, may later lose capacity (e.g. due to acute delirium).

**Figure 1.**
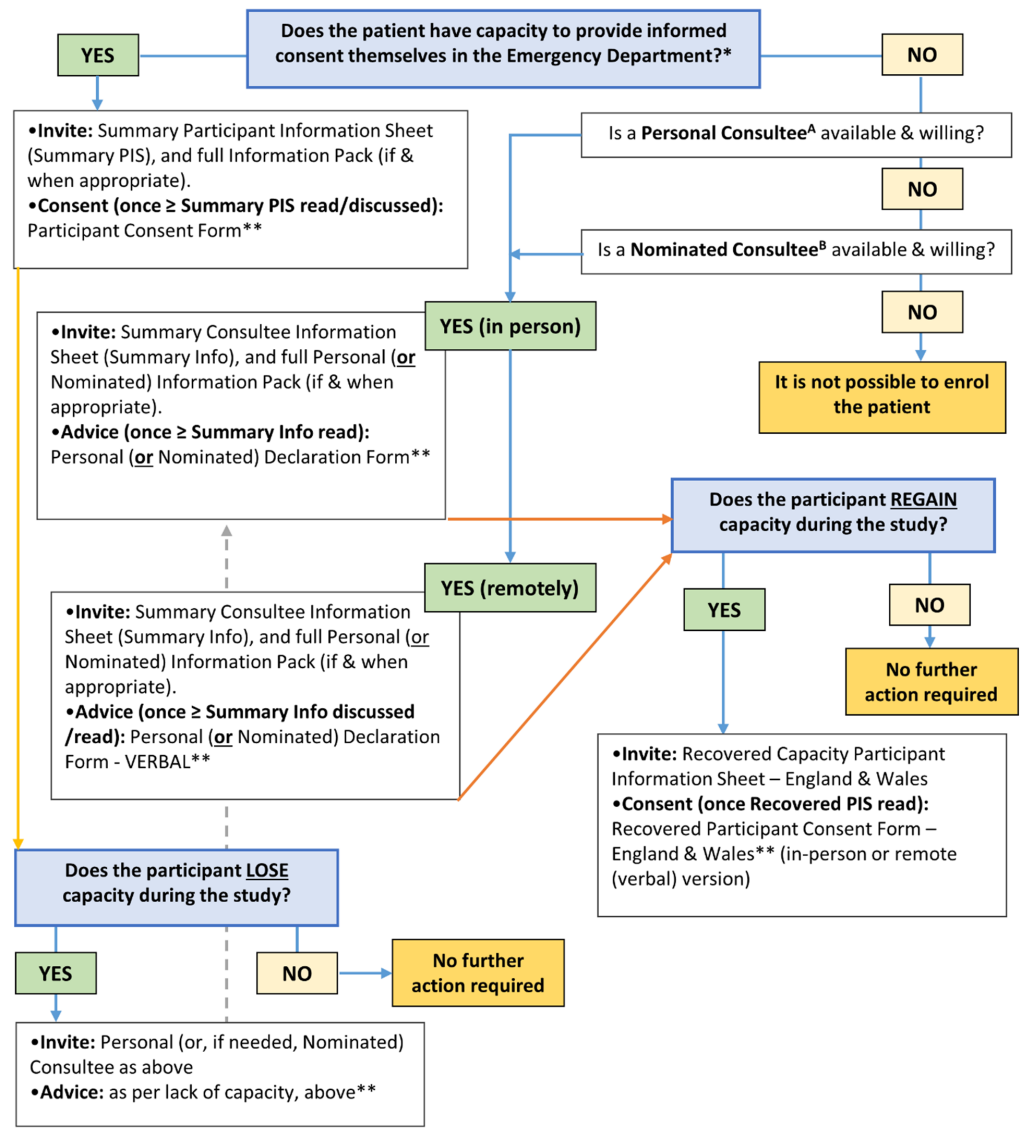
Participant information and consent pathways for patients in England and Wales. *Trial invitation and consent processes are in line with the Mental Capacity Act 2005 for patients in England and Wales. **The default method of completion is via the appropriate online eConsent (or eDeclaration) Form. If the online form is not feasible, then a paper version is available. Site Staff should update the RELIEF Screening Log at all relevant timepoints. A Personal Consultee. The patient’s partner, or a particular friend or carer who is not seeking renumeration for doing so or acting in a professional capacity. B Nominated Consultee. Clinician at site appointed by PI; may include a member of the care team as long as they are not connected with the project to avoid potential conflict; the CI and locals PIs are therefore excluded.

Participant information and consent pathways for those patients who may lack or lose capacity to consent within this trial are complex and will vary according to local legal processes. In England and Wales, this process is guided by the Mental Capacity Act 2005 and pathways are summarised in Figure 1.

### Randomisation

Immediately after diagnosis in the ED participants will be randomised on a 1:1 ratio to either the lidocaine patch (intervention) or standard care (control) group. An online randomisation system will be used, with the randomisation sequence being generated by the company “Sealed EnvelopeTM” (London, United Kingdom). Randomisation will be stratified by both trial site and gender as a dichotomous variable and blocked within strata.

### Intervention (lidocaine patch)

Patients randomised to the intervention group will have up to 3 × 700mg lidocaine patches (Ralvo®) applied over the most painful area of rib injury within the ED. The patches will be applied once daily for 12 hours in accordance with the manufacturer’s (Grünenthal, Aachen, Germany) instructions, followed by a 12-hour patch-free period. Treatment will continue for up to 72 hours or until the time of discharge, whichever is sooner. The 72 hour intervention period was chosen based on a prior randomised efficacy trial ^14^ and aligns to local practices within the host site. Trial medication will be obtained from local pharmacy supplies and not supplied by the trial. The intervention is additive to standard clinical management.

### Control (standard clinical management)

All patients regardless of randomised treatment allocation will receive standardised treatment according to local analgesic guidelines for patients with rib fractures. This should include prescription of regular paracetamol, ibuprofen (unless contraindicated) and codeine phosphate (or alternative weak opioid). Adherence to prescribing according to local guidelines, collected from all sites, will be recorded. It is anticipated that some participants may require stronger opioid analgesia, such as oral morphine. Opioid prescription will not be controlled and will be analysed for potential differences between groups and will include patient controlled analgesia. Some recruited participants will also undergo regional anaesthesia such as thoracic epidural or nerve block. Should regional anaesthesia be deemed necessary by the treating clinician, patches will be removed, no further patches will be applied, and group allocation maintained (data will be collected on advanced analgesia use).

### Clinical procedures

Trial participants will have undergone the standard clinical assessment and treatment of patients with suspected rib fractures in the ED. This includes a triage, routine initial observations of pulse, blood pressure, respiratory rate, and oxygen saturations.

***Analgesia.*** Prior to trial screening, trial participants will be offered standard analgesia according to local practice by clinical staff. This practice will not be altered for trial purposes. Data on analgesia provision prior to trial screening, in participating patients, will be collected retrospectively via medical notes to ensure no differences exist between groups.

***Imaging.*** Imaging to identify the presence of rib fractures will be entirely at the discretion of the treating clinician and not altered for trial purposes. Appropriate imaging modalities to identify the presence of rib fractures will include chest X-Ray and CT scan, these will not be performed outside of standard care.

## Outcomes

### Feasibility outcomes

Feasibility outcomes together with progression criteria are summarised in Table 1.

**Table 1.** Feasibility parameters required to progress to a full trial.

	Participants	Anticipated action
**Go (Green)**	• **Recruitment:** >70% of expected recruitment. • **Follow Up:** ≥75% of data for suggested primary outcome of 30-day pulmonary complications. • **Adherence:** ≥75% adherence to the intervention.	Continue to main trial.
**Amend (Amber)**	• **Recruitment:** 50–70% of expected recruitment target. • **Follow Up:** 65–74% of data for suggested primary outcome of 30-day pulmonary complications. • **Adherence:** 65–74% adherence to the intervention.	Identify remediable factors, discuss with TMG and TSC.
**Stop (Red)**	• **Recruitment:** <50% of expected recruitment target. • **Follow Up:** <65% of data for suggested primary outcome of 30-day pulmonary complications. • **Adherence:** <65% adherence to the intervention.	Do not progress to main trial, unless there is a strong case that unanticipated remediable factors have been identified and can be addressed after further discussion with the funder.

### Definitions of feasibility parameters

**Recruitment:** Expected recruitment will take place within an 18 month recruitment window

**Follow-up:** The follow-up parameter looks at data completeness of 30-day pulmonary complications; this is a binary data point of No complications vs Any (at least one) complications. The cut-off of 75% for Go (Green) was agreed with the trial funder, however we aim to achieve as close to 100% data completeness as possible for the primary outcome.

**Adherence:** To assess the feasibility parameter of adherence, we will consider only the time that the participant is in the Emergency Department (ED). For a participant in the intervention arm, they would be considered adherent if at least one lidocaine patch was applied whilst they were in the ED. For a participant in the control arm, they would be considered adherent if no lidocaine patches were applied whilst they were in the ED.

### Clinical outcomes

Clinical outcomes are summarised in Table 2. Clinical outcomes are collected to understand feasibility of data collection to inform a definitive trial and not to conduct hypothesis testing.

**Table 2.** Schedule of trial assessments and key participant-related procedures.

Data collection timepoint (→) Data capture / key trial procedure (↓)	In the Emergency Department (ED)		Post-Randomisation Follow-up		
	Recruitment	Post-Recruitment (Baseline)	72-hours (3-days) Intervention Period	30-days (+10 days)	Up to 90-days
Screening, Consent & Randomisation	**X**				
Case Report Forms (CRFs: trial-specific)		**X**	**X**	**X**	
Observations and Injury data * **^A^**		**X**			
Rockwood Frailty Scale *		**X**			
EQ-5D-5L (retrospective pre-injury, and post injury at Baseline)		**X**		**X ^B^**	
Immobility: Timed Up and Go Test		**X**	**X**		
Intervention delivery		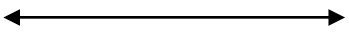			
Total opioid consumption (first 72 hours) * ^D^			**X**		
Pain: 4-hourly VAS and Abbey Pain Scores (during first 72 hours)		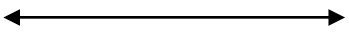			
Delirium: Daily 4-AT during 72-hour intervention period. Single item question at 30-days.		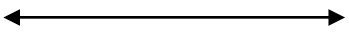		**X**	
Opioid Induced Constipation (notes screen for Bristol Stool Chart) *			**X**		
Pulmonary complications (notes screen) and Intensive Care Unit admission *				**X**	
Mortality (notes screen) *				**X**	
Hospital Re-admission (GP records/notes screen) *				**X**	
Length of Stay *				**X**	
Discharge destination and support required *				**X**	
Chest Trauma Score (RibPROM)				**X ^B^**	
ICECAP-O Quality of Life				**X ^B^**	
Qualitative interviews **^C^**		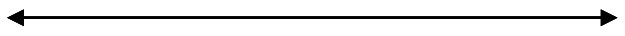			

* These data are routinely collected, thus are not collected specifically for the purpose of this trial. Data will be taken from medical records and recorded in the CRF(s). A Injury Severity Score (ISS) may be calculated retrospectively after validation by the Trauma and Audit Research Network. B Face-to-face (or online) data collection if still inpatient, or via post/telephone/online if not (+10 days allowance). C Qualitative interviews with patients (participants) and people with carer responsibilities (e.g. Personal Consultees, or other subsequent carers) will take place, noting that timing is flexible from baseline and up to 90 days after initial ED attendance (randomisation). D Total Opioid Consumption: Data will be presented as a continuous variable measure of central tendency of dose by each drug (separately by route, ie Drug A intravenous, Drug A oral, Drug B intravenous etc). Then a variable will be generated that represents total oral morphine equivalent for each participant, and this will be presented via a measure of central tendency and dispersion appropriate to the nature of the distribution.

### Data collection and follow-up

Demographic and clinical data will be recorded after randomisation by the treating clinician or member of the trial team. Data will be collected face-to-face (where the participant is still an inpatient, or via telephone, post and/or online if not) or from patient records using routinely collected data. Table 2 presents a schedule of trial assessments (SPIRIT 2013).

***Baseline data.*** Participants will be asked to complete the baseline trial questionnaire which contains the EQ-5D-5L ^22^ to capture both retrospective pre-injury and baseline post injury health. Research staff will complete a baseline case report form (CRF) to capture information such as demographics, injury details, relevant past medical history and frailty ^23^.

***72-hour data collection (intervention period).*** Participants will be asked to complete a questionnaire booklet over the course of 72 hours post-randomisation (or up until point of discharge if sooner) which contains 4-hourly pain assessment using a Visual Analogue Scale (VAS) (scaled from 0-100) along a 100mm horizontal line with verbal anchors at each end of ‘no pain’ and ‘worst pain possible’. Participants will be provided with the questionnaire booklet and asked to report pain scores every 4 hours, with periods of sleep retrospectively reported. Where necessary, the scores may be completed by a member of the research or clinical teams. Questionnaire completeness will be collected and reported as a percentage.

Researchers will complete a CRF over the course of 72 hours post randomisation. This CRF will contain the following: the Abbey pain scale (designed for use in non-verbal patients with dementia ^24^), total opioid consumption in the first 72 hours of attendance (including patient controlled analgesia), treatments received (if cross-over occurs then details, including reason(s) why, should be recorded), assessment of 4-AT brief clinical instrument for delirium detection ^25^, timed Up and Go Test at baseline (day 1) and 72 hours on completion of the intervention period ^26^, opiate induced constipation and adverse events.

***30 day data collection.*** Participants (and/or person with caring responsibility, where applicable) will be contacted and asked to complete a questionnaire booklet (postal paper booklet or online equivalent if preferred) containing the EQ-5D-5L, ICECAP-O and Chest Trauma Score ^22, 27, 28^.

Researchers will complete a 30 day CRF from information gathered from medical notes to capture: pulmonary complications, development of delirium, mortality, hospital discharge status, re-admission, length of stay, hospital resource use and adverse events.

### Sample size and analysis plan

As this is a feasibility trial, a formal sample size calculation based on statistical power to detect a specified treatment effect size is not appropriate. In line with published “rules-of-thumb” we have determined that a total sample size of 100 (50 per arm) will be sufficient to provide estimates of recruitment, retention, data completion and adherence ^29^.

The analysis will focus on reporting feasibility measures (eligibility, recruitment, retention and data completeness). Data will be analysed and reported following the CONSORT guidance extension to feasibility studies and will include a CONSORT flow diagram, descriptive and summary statistics both overall and by treatment arm ^18, 19^.

This trial is not powered to carry out hypothesis testing. Descriptive statistics for the patient characteristics and clinical outcome data will also be reported overall and by treatment group; as means or medians with measures of dispersion for continuous outcomes (as appropriate given the form of their distribution) and frequencies and percentages for categorical outcomes.

The suggested primary outcome for a definitive trial is a binary measure of pulmonary complications (subject to change)-NO complications vs ANY complications. Pulmonary complications collected are; Type 1 respiratory failure, Type 2 respiratory failure, pulmonary embolism, pneumonia, ventilator associated pneumonia, adult respiratory distress syndrome, lower respiratory tract infection (non pneumonia), new pleural effusion >24 hours after injury, empyema, COVID-19 pneumonitis and other respiratory complication.

### Integrated qualitative study

To explore details of the trial design, acceptability of proposed outcome measures to patients and understand recruitment processes for the main trial, up to 24 participants and 6 Personal Consultees with carer responsibilities, who agreed to further contact during initial consent (in a ratio of 2:1 intervention:control, from at least 3 sites) will be invited to take part in a semi-structured interview with a qualitative researcher. A purposive sample will be selected to reflect maximum variation in socio-demographics, age, and ethnicity. In addition, two focus groups with healthcare professionals from at least two sites will be undertaken to evaluate their experiences of treatment and views of trial processes, including evaluating equipoise around the use of the intervention.

### Health economic scoping

An evaluation of the feasibility of identifying and measuring health economics outcomes data will be completed as part of the trial. As this is a feasibility trial, the focus will be on establishing the most appropriate outcome measures for inclusion in a future economic evaluation alongside the definitive trial.

### Safety and data governance

Serious and other adverse events (S/AEs) will be recorded and reported in accordance with the GCP guidelines and the Sponsor’s Research Related Adverse Event Reporting Policy. Participant safety will be monitored by the Trial Management Group, Sponsor and Trial Steering Committee and the trial will be stopped if any indication of harm from using the intervention is found. Lidocaine patches have been used since 1999 for the treatment of shingles. Numerous studies have evaluated safety of the patches and no clinically significant systemic adverse effects have been noted, including when used in an elderly population in high doses ^11^. Therefore, we do not anticipate any unexpected SAEs in relation to the intervention.

All administrative and clinical trial data will be stored in a REDCap (Vanderbilt University, USA) database. REDCap is a secure, web-based electronic data capture (EDC) system designed for the collection of research data. North Bristol NHS Trust and the Bristol Trials Centre are joint data controllers for the RELIEF trial. Data will be held at the University of Bristol and will conform to the University of Bristol Data Security Policy and in Compliance with the General Data Protection Regulation (GDPR) as it applies in the UK, tailored by the Data Protection Act 2018.

## Dissemination

A writing committee will be established which will be responsible for preparing scientific reports of the trial findings. The aim will be to publish a primary manuscript in a general medical journal, published as open access, with additional qualitative analyses described in specialty journals. A full report will be completed for the funder. 

## Patient and Public Involvement

The Older Person’s Trauma patient group from the Severn Major Trauma Network was key in identifying the unmet need for improved analgesia in older patients with chest trauma and assisted in developing the research question through their own experiences of being admitted to hospital following injury.

In addition, we developed the protocol with the assistance of the RELIEF Patient Advisory Group (PAG) who will continue to assist in refinement of the protocol and research processes throughout the duration of the trial. During initial protocol development, in depth focus groups with our PAG allowed us to explore issues specific to trials recruiting older injured patients. These included, but were not limited to; the acceptability of eligibility criteria (the use of a binary age cut-off of 65 years), the acceptability of randomisation and consent processes (personal consultee assent in patients with cognitive impairments to ensure these patients could be included), the development of outcome measures (highlighting the importance of constipation as a medication side effect), and the acceptability of follow-up processes (paper questionnaires to avoid digital exclusion in this population). Our PAG reviewed, edited and approved all patient facing materials and ensured modifications were made for older patients such as readability/large print and the option to mark an “X” on the consent form for patients who may have difficulty in marking their initials. PAG members are included on the TMG and an independent public member will sit on the TSC. The PAG will be key to informing the progression to a full trial following feasibility assessment, which will be decided upon completion of this feasibility trial, and will agree plans for dissemination.

## Study status

Recruitment to the RELIEF trial is now complete. Trial data is being cleaned in preparation for analysis and reporting.

## Discussion

The Randomised Evaluation of early topical Lidocaine patches In Elderly patients admitted to hospital with rib Fractures (RELIEF) feasibility trial will determine whether a definitive trial is feasible and to optimise the design of such a trial. Beyond the uncertainties around conducting such a trial with older patients with rib fractures, in an emergency setting, which necessitate a feasibility trial, there are additional clinical and operational challenges that should be considered.

Clinical data collected within this trial are extensive and likely to be optimised. Whilst the likely primary outcome for a definitive trial will be the development of pulmonary complications, this will require careful consideration with stakeholders should the definitive trial prove feasible. There is increasing acknowledgement of a disconnect between patients, healthcare professionals and trialists in their choice of primary outcome in clinical trials ^30^. Our data collection, which includes pain, opioid use, constipation, medication side effects and patient reported outcomes will therefore afford us to explore this issue further within both our integrated qualitative study and with further patient and public involvement to inform definitive trial design.

We have used a binary age cut-off of 65 years for recruitment to this feasibility trial. Age has traditionally been used as a predictor of adverse outcomes in patients with trauma ^1^. However, age is a heterogenous state and does not describe an individual’s intrinsic fitness. There is emerging evidence that frailty, a state of reduced physiological and cognitive reserve, as measured by the Clinical Frailty Scale, may offer a more exquisite predictor of adverse outcomes in patients with serious injuries ^31^. Collecting frailty data within this RCT affords the opportunity to alter definitive trial design to include those patients who stand to benefit most from our trial intervention.

Inpatient teams in some sites may utilise lidocaine patches as part of standard care. Whilst we are testing lidocaine patches as an early intervention, applied within the ED in the immediate phase after injury, recording subsequent lidocaine patch use in an inpatient setting will allow us to understand issues with equipoise and crossover that may necessitate the use of placebo patches within a definitive trial.

Undertaking this feasibility trial will afford understanding of additional operational issues, out-with our predetermined feasibility outcomes. These include optimisation of patient facing documents for our elderly population, such as the use of large print or facilitating consent using a mark instead of initials, provision and design of questionnaires for patients with limited digital access and a greater understanding of inpatient pathways and analgesia delivery in this vulnerable patient group.

Interventions to improve clinical outcomes in elderly patients with rib fractures are urgently required to enhance patient care. This feasibility trial will test a novel early intervention which has the potential of fulfilling this unmet need.

## Ethics approval

The protocol (Version 4.0 04/03/2022) and other related participant facing documents have been approved by the UK Health Research Authority and U.K Research Ethics Committees (REC): 21/SC/0019 (South Central – Oxford C REC; IRAS reference 285096; Favourable Opinion 30 March 2021) and 21/SS/0043 (Scotland A REC; IRAS reference and 299793; Favourable Opinion 08 July 2021).

## Data Availability

No underlying data are associated with this article. Further information and patient facing materials (including model consent forms) are available at https://relief.blogs.bristol.ac.uk/. Figshare: SPIRIT checklist for ‘The Randomised Evaluation of early topical Lidocaine patches In Elderly patients admitted to hospital with rib Fractures (RELIEF): feasibility trial protocol‘ https://doi.org/10.6084/m9.figshare.23545998. Data are available under the terms of the Creative Commons Attribution 4.0 International license (CC-BY 4.0).
