# Supplementary material for: The Randomised Evaluation of early topical Lidocaine patches In Elderly patients admitted to hospital with rib Fractures (RELIEF): feasibility trial protocol
Source: NIHR Open Res. 2023 Sep 25;3:38. Originally published 2023 Jul 27. [Version 2] doi: 10.3310/nihropenres.13438.2 (PMC10593328; doi:10.3310/nihropenres.13438.2)
Supplement: Peer review history

# Reviewer report: Reviewer response for version 2

**DOI:** 10.3310/nihropenres.14641.r30447

**Published:** 2023-10-05

**Authors:** Eleonora Balzani

**Affiliations:** 1 University of Turin, Torino, Italy

I thank the authors for considering my revisions. Although not all the points I highlighted have been addressed, I believe the protocol is robust enough for publication. Therefore, I have no further comments to make.

Is the study design appropriate for the research question?

Yes

Is the rationale for, and objectives of, the study clearly described?

Yes

Are sufficient details of the methods provided to allow replication by others?

Partly

Are the datasets clearly presented in a useable and accessible format?

No

Reviewer Expertise:

Regional anesthesia, pulmonary disease

I confirm that I have read this submission and believe that I have an appropriate level of expertise to confirm that it is of an acceptable scientific standard.

# Reviewer report: Reviewer response for version 2

**DOI:** 10.3310/nihropenres.14641.r30446

**Published:** 2023-10-05

**Authors:** Abhijit Nair

**Affiliations:** 1 Ibra Hospital, Ibra, Oman

The authors have made amendments based on the comments raised in the previous review report. In its present form, the submission can be accepted.

Is the study design appropriate for the research question?

Partly

Is the rationale for, and objectives of, the study clearly described?

Partly

Are sufficient details of the methods provided to allow replication by others?

Partly

Are the datasets clearly presented in a useable and accessible format?

Yes

Reviewer Expertise:

Anesthesiology, Acute Pain, Regional Anesthesia, Perioperative Care

I confirm that I have read this submission and believe that I have an appropriate level of expertise to confirm that it is of an acceptable scientific standard.

# Reviewer report: Reviewer response for version 1

**DOI:** 10.3310/nihropenres.14576.r30065

**Published:** 2023-09-07

**Authors:** Abhijit Nair

**Affiliations:** 1 Ibra Hospital, Ibra, Oman

Dear authors,

 I have gone through your submission, and I have a few concerns about it. Kindly go through the comments below.

 There are several scores to quantify rib fracture. I suggest the authors should use one of the validated scoring systems to enroll the patients to make it standardized.

 (Fokin A, Wycech J, Crawford M, Puente I. Quantification of rib fractures by different scoring systems. J Surg Res. 2018 Sep;229:1-8)

 It needs to be explicitly mentioned in the inclusion criteria that patients who receive chest wall blocks- serratus anterior plane block, erector spinae plane block, paravertebral block, or a thoracic epidural will be excluded from inclusion, in either group.

 The authors have mentioned: Patients randomised to the intervention group will have up to 3 × 700mg lidocaine patches (Ralvo®) applied over the most painful area of rib injury within the ED. What if the 3 patches do not cover all the areas, especially in the presence of bilateral rib fractures?

 The authors have mentioned that patients having chest tubes will also be included. It is well known that the chest tube insertion site is extremely painful and there are high chances that the patient could point that as very painful and thus patch will be placed. This could add a lot of bias and inappropriate treatment.

 For a study involving an analgesic modality (lidocaine patch), analgesic efficacy in the form of opioid consumption or pain scores at various time intervals would have been a better primary outcome instead of a very vague outcome in the form of yes/no.

Is the study design appropriate for the research question?

Partly

Is the rationale for, and objectives of, the study clearly described?

Partly

Are sufficient details of the methods provided to allow replication by others?

Partly

Are the datasets clearly presented in a useable and accessible format?

Yes

Reviewer Expertise:

Anesthesiology, Acute Pain, Regional Anesthesia, Perioperative Care

I confirm that I have read this submission and believe that I have an appropriate level of expertise to state that I do not consider it to be of an acceptable scientific standard, for reasons outlined above.

## References

[1] : Quantification of rib fractures by different scoring systems. *J Surg Res* .2018;229: 10.1016/j.jss.2018.03.025 1-8 10.1016/j.jss.2018.03.025 29936974

# Reviewer report: Reviewer response for version 1

**DOI:** 10.3310/nihropenres.14576.r30071

**Published:** 2023-09-07

**Authors:** Eleonora Balzani

**Affiliations:** 1 University of Turin, Torino, Italy

1. "T0": Please provide a clear definition of "T0" in the study, specifying whether it refers to the patient's arrival at the emergency department or to the initial assessment by the physician. Is there an evaluation of the time elapsed between the trauma, arrival at the emergency department, and the assessment of eligibility criteria by the designated personnel? Additionally, kindly specify the time frame allocated for patient enrollment in the study from the emergency department admission.
2. Definitions of complications: Precise and detailed definitions are required for each type of complication, such as Hospital-Acquired Pneumonia and Acute Respiratory Distress Syndrome. It is essential to include specific diagnostic criteria and relevant symptoms to identify these complications during the study.
3. Placement of lidocaine patch: In the event of an open wound with reported maximum pain in the area, please outline the protocol for patch placement.
4. Management of control group patients with regional blocks: Provide a comprehensive description of how control group patients undergoing regional blocks, such as paravertebral or erector spinae plane blocks, will be managed within the study. Specify whether such patients will be excluded or how they will be considered for analysis.
5. Monitoring of adverse effects: Please delineate the monitoring plan for adverse effects, such as cardiac block. Consider the utilization of postoperative monitoring, such as heart rate, as an indirect parameter to evaluate patient pain and intervention safety.
6. Additional analyses: Evaluate the feasibility of conducting subgroup analyses, for example, comparing patients with prior chronic opioid therapy to opioid-naive individuals. I recommend adopting the classification found in the paper DOI: 10.1213/ANE.0000000000004018 to quantify patients' prior exposure. Additionally, consider conducting an ad hoc analysis for patients with dementia or communication difficulties for further investigation.
7. Intent-to-treat (ITT) analysis: Describe the plan for the ITT analysis and how non-adherence to the protocol and missing data will be addressed, for instance, using randomization and multiple imputation techniques.
8. Informed consent: Include informed consent as an appendix to ensure that participants are fully informed and have given voluntary informed consent before participating in the study.
9. Appendix with the dataset: Please provide an appendix containing the dataset with measured variables, specifying the timing of measurement and the evaluation conducted for each variable (e.g., heart rate, ECG monitoring, presence of HAP, chest radiography).

Is the study design appropriate for the research question?

Yes

Is the rationale for, and objectives of, the study clearly described?

Yes

Are sufficient details of the methods provided to allow replication by others?

Partly

Are the datasets clearly presented in a useable and accessible format?

No

Reviewer Expertise:

Regional anesthesia, pulmonary disease

I confirm that I have read this submission and believe that I have an appropriate level of expertise to confirm that it is of an acceptable scientific standard, however I have significant reservations, as outlined above.

## References

[1] : American Society for Enhanced Recovery and Perioperative Quality Initiative Joint Consensus Statement on Perioperative Management of Patients on Preoperative Opioid Therapy. *Anesth Analg* .2019;129(2) : 10.1213/ANE.0000000000004018 553-566 10.1213/ANE.0000000000004018 30768461
